# Translocator Protein 18 kDa (TSPO): A Promising Molecular Target for Image-Guided Surgery of Solid Cancers

**DOI:** 10.34172/apb.2024.015

**Published:** 2023-10-14

**Authors:** Hendris Wongso, Ahmad Kurniawan, Yanuar Setiadi, Crhisterra E. Kusumaningrum, Eva M. Widyasari, Teguh H.A. Wibawa, Isa Mahendra, Muhamad B. Febrian, Maula E. Sriyani, Iim Halimah, Isti Daruwati, Rudi Gunawan, Arifudin Achmad, Dwianto H. Nugraha, Ronny Lesmana, Ari S. Nugraha

**Affiliations:** ^1^Research Center for Radioisotope, Radiopharmaceutical, and Biodosimetry Technology, Research Organization for Nuclear Energy, National Research and Innovation Agency Republic of Indonesia, Puspiptek, Banten 15314, Indonesia; ^2^Research Collaboration Center for Theranostic Radiopharmaceuticals, National Research and Innovation Agency, Jl. Ir. Soekarno KM 21, Jatinangor 45363, Indonesia; ^3^Research Center for Environmental and Clean Technology, Research Organization for Life Sciences and Environment, National Research and Innovation Agency, Puspiptek, Banten 15314, Indonesia; ^4^Department of Pharmaceutical Analysis and Medicinal Chemistry, Faculty of Pharmacy, Universitas Padjadjaran, Jl. Ir. Soekarno KM 21, Jatinangor 45363, Indonesia; ^5^Department of Nuclear Medicine and Molecular Theranostics, Faculty of Medicine, Universitas Padjadjaran, Bandung 40161; ^6^Oncology and Stem Cells Working Group, Faculty of Medicine, Universitas Padjadjaran, Bandung 40161; ^7^Technology Development Division, PT. Kalbe Farma, Jakarta, Indonesia; ^8^Department of Biomedical Science, Faculty of Medicine, Universitas Padjadjaran, Jatinangor 45363, Indonesia; ^9^Physiology Molecular, Division of Biological Activity, Central Laboratory, Universitas Padjadjaran, Jatinangor 45363, Indonesia; ^10^Laboratory of Sciences, Graduate School, Universitas Padjadjaran, Bandung, Indonesia; ^11^Drug Utilisation and Discovery Research Group, Faculty of Pharmacy, Universitas Jember, Jember 68121, Indonesia; ^12^School of Chemistry and Molecular Biosciences, Molecular Horizons, University of Wollongong, Wollongong, New South Wales, 2522, Australia

**Keywords:** Cancers, Image-guided surgery, Molecular target, Translocator protein 18-kDa (TSPO)

## Abstract

The translocator protein 18-kDa (TSPO) is a mitochondrial membrane protein that is previously identified as the peripheral benzodiazepine receptor (PBR). Furthermore, it plays a significant role in a diverse range of biochemical processes, including steroidogenesis, mitochondrial cholesterol transport, cell survival and death, cell proliferation, and carcinogenesis. Several investigations also reported its roles in various types of cancers, including colorectal, brain, breast, prostate, and lung cancers, as well as melanoma. According to a previous study, the expression of TSPO was upregulated in cancer cells, which corresponds to an aggressive phenotype and/or poor prognosis. Consequently, the potential for crafting diagnostic and prognostic tools with a focus on TSPO holds great potential. In this context, several radioligands designed to target this protein have been identified, and some of the candidates have advanced to clinical trials. In recent years, the use of hybrid probes with radioactive and fluorescence molecules for image-guided surgery has exhibited promising results in animal and human studies. This indicates that the approach can serve as a valuable surgical navigator during cancer surgery. The current hybrid probes are built from various molecular platforms, including small molecules, nanoparticles, and antibodies. Although several TSPO-targeted imaging probes have been developed, their development for image-guided surgery of cancers is still limited. Therefore, this review aims to highlight recent findings on the involvement of TSPO in carcinogenesis, as well as provide a new perspective on the potential application of TSPO-targeted hybrid probes for image-guided surgery.

## Introduction

**Figure 1:**
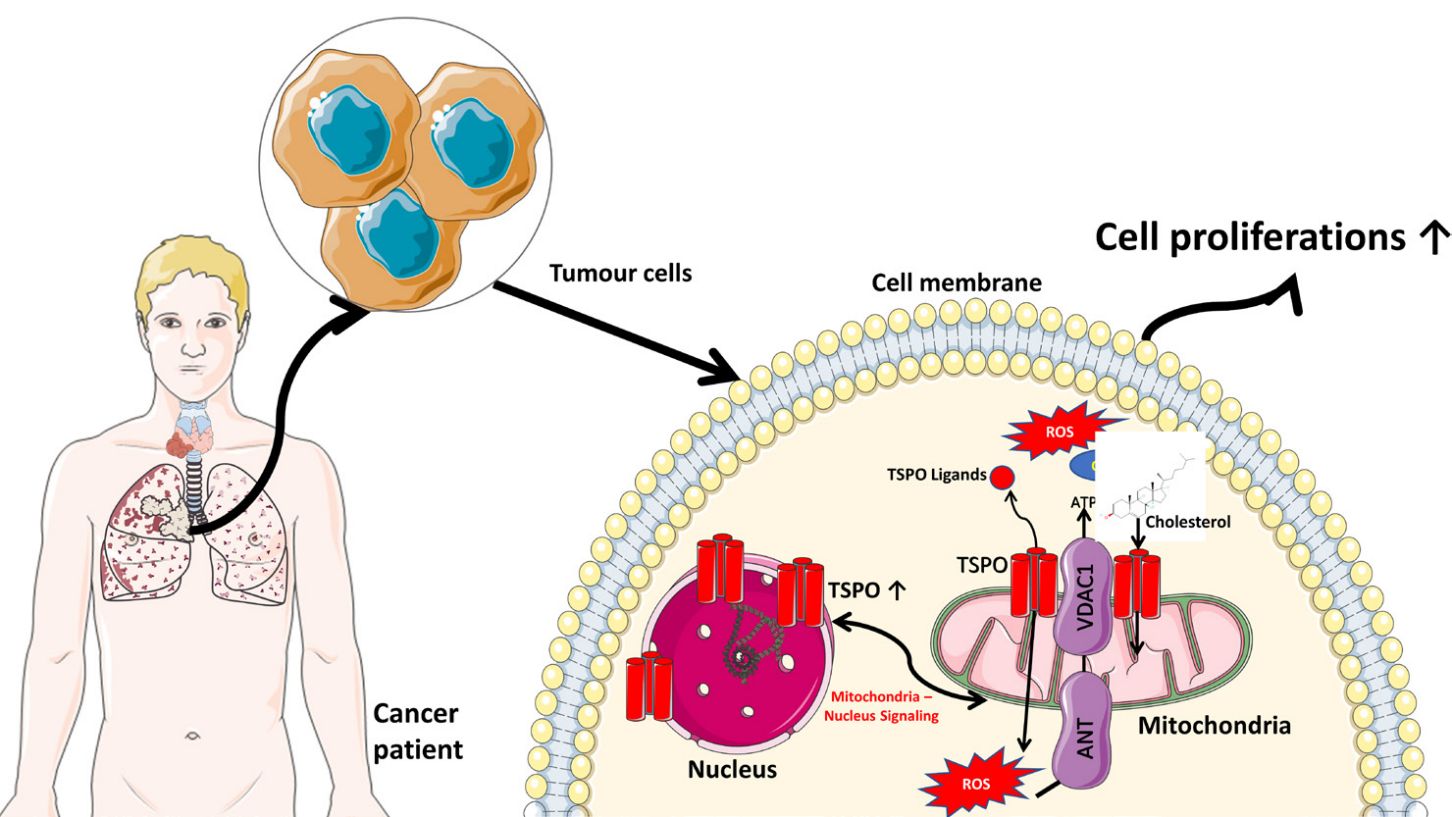
The molecular mechanism of TSPO in tumor cells. TSPO is primarily located on the outer mitochondria membrane, which has a role in transporting cholesterol from the outer to the inner mitochondrial membrane during the steroidogenesis process. TSPO is also involved in Ca^2+^ ion regulations by working together with VDAC1 (voltage-dependent anion channel 1), ANT (adenine nucleotide transporter), and ATPase for ROS generation and ATP production.^9^ The increase of TSPO in tumor cells also provides energy for cell proliferation.^8^ Several images were taken from Servier Medical Art: smart.servier.com (Creative Commons Attribution 3.0 unported license)

 The translocator protein 18-kDa (TSPO), previously known as the peripheral benzodiazepine receptor (PBR), is a ubiquitous transmembrane protein predominantly located in the outer mitochondrial membrane (Figure 1) and in the glial cells of the brain.^1^ Furthermore, it connects with the mitochondrial permeability transition pore and shows high-affinity binding to cholesterol and various ligands.^2,3^ This protein exhibits a wide range of imbalances within the central nervous system (CNS) and rapidly becomes activated in response to pathological events.^4,5^ The roles, expression, and pharmacology properties of TSPO have been extensively investigated in several studies, specifically drug-binding interactions.^6^ Several studies also showed that TSPO played significant roles in different processes, such as cell proliferation in normal and malignant tissues, steroidogenesis, and apoptosis. According to recent studies, it is also associated with innate immune response, the formation and regulation of reactive oxygen species (ROS), and mitochondrial energy metabolism.^7,8^ TSPO has also been linked with various human disorders, including mood disorders, anxiety, stress, brain injury, and neurodegenerative diseases.^9^ However, its overexpression in carcinogenesis has sparked considerable interest as a potential biological marker and a promising molecular target in chemotherapy.^10^

 TSPO is present in nearly all tissues, but it exhibits significant variation in expression.^11^ In normal brain, microglia show limited expression. However, increased concentration is often observed after brain injury, with upregulation in neurodegenerative disorders.^12^ Several reports showed that increased TSPO levels were well-documented in the field of oncology, and had been associated with the disease development and malignancy in breast, brain, and prostate cancer.^13^ The protein also experiences substantial upregulation in steroidogenic cells, including adrenocortical, testicular, brain glial tumor cells, and other tumor cells of the brain, colon, and ovary.^11^ Due to its overexpression in several cancerous tissues, TSPO presents an intriguing subcellular target in oncology, specifically for staging disease conditions and therapeutic purposes. At present, several radioactive ligands have been developed for imaging TSPO expression, biodistribution in normal physiological and pathological states, and measuring the correlation between expression and disease progression.^14-17^ [^11^C]PK11195 was the first generation of radioligand for TSPO, which was developed more than two decades ago and has been widely used for monitoring neuroinflammation in numerous neurodegenerative diseases. However, this tracer possesses a relatively low signal-to-noise ratio due to off-target binding.^15^ The latest discovery of third-generation TSPO radioligands has largely addressed the limitations of the first and second-generation compounds in clinical studies.^17^

 The primary objective of surgery is to achieve a complete resection of all tumor cells and tissues to attain a negative surgical margin. However, this remains a formidable challenge, despite the extensive use of preoperative imaging modalities during surgical procedures, such as single-photon emission tomography (SPECT) and positron emission tomography (PET). These modalities are inadequate for the intraoperative detection of tumors (guiding tumor resection). This indicates that the persistently high local tumor recurrence rates are still a significant concern after the surgery.^18,19^ Based on this result, there is a pressing need to develop more reliable molecular probes that target specific receptor(s) involved in carcinogenesis, such as the development of TSPO-based molecular probes.

 Due to its overexpression in tumor lesions, TSPO offers diverse applications in pharmacological studies, specifically in the field of oncology and nuclear medicine. One notable application is its potential as a promising molecular target in optical- and radio-based guided surgery of solid cancers. Cohen et al developed combined imaging based on TSPO-targeted PET and optical probes for visualizing pre-malignant and malignant pancreatic lesions. The study showed that the combined PET and fluorescence agents could be used for image-guided surgery.^20^ Although TSPO has been linked to a variety of malignancy conditions, the study on the potential use of this protein as a targeted biomarker during surgical procedures of cancers is still limited.

 Recent advancements in surgical oncology have introduced various techniques for conducting cancer surgery, including radio-guided surgery (RGS),^21^ fluorescence image-guided surgery (FIGS),^22^ or a combination of both approaches (hybrid radionuclide-optical imaging).^23^ RGS involves the surgeon using a handheld radiation monitor to navigate and remove solid cancers that are labeled with a radioactive substance. This technique helps to distinguish between cancerous and normal tissues, as well as to verify clear surgical margins. RGS relies on the use of *γ* emitting tracers (administrated either systemically or locoregionally), and there has been developing interest in the use of *β* - emitting tracers.^24,25^ Over the past decade, several significant improvements in RGS technologies have been achieved, particularly the gamma probes, dual-modality handheld gamma cameras, and medical robotic equipment combined with augmented reality technology. Furthermore, more sensitive techniques employing Cerenkov radiation, also referred as Cerenkov luminescence imaging (CLI), have been introduced.^26^

 Optical imaging that employs the FIGS technique has the potential to revolutionize cancer surgery by minimizing unnecessary damage to normal tissues.^27,28^ FIGS has been used for intraoperative optical cancer imaging, where surgeons use fluorescence compounds to visualize cancerous tissues, define tumor-positive margins, and identify metastases.^29,30^ At present, several FIGS agents based on macromolecules (antibodies), peptides, and small molecules have been developed, and some of the candidates have progressed to clinical trials. These agents were designed to target several molecular biomarkers, such as epidermal growth factor receptor (EGFR), vascular endothelial growth factor receptor (VEGFR),^31^ gastrin-releasing peptide receptor (GRPR),^32^ somatostatin receptor 5 (SSTR5),^33^ and the cluster of differentiation 24 (CD-24).^34^

 Although RGS and FIGS methods have experienced significant growth in recent decades, with a broad number of targeted receptors being studied, the exploration of TSPO as a potential molecular target in surgical oncology is still limited. Therefore, this review aims to highlight recent results on the involvement of TSPO in various types of cancers and discuss its prospect as a novel targeted receptor in cancer surgery.

## Clinical implications of TSPO

 Microglia, a type of glial cell (neuroglia), were the most abundant resident macrophage population (histiocytes) in the CNS. These macrophages played a substantial role in maintaining and establishing the nervous system.^35-37^ Furthermore, they were widely distributed in the brain, constituting nearly 10% of the total cell populations within the organ.^38^ As part of the immune cells, microglia responded to any change and imbalance, including brain injury or diseases. External stimuli, such as pathogens, infections, trauma, stroke, and degenerative disorders, facilitated the production of proinflammatory substances, chemokines, and cytokines, which eventually activated microglia.^39^ During CNS imbalance, microglia were often transformed from a resting phenotype to an activated variant.^40,41^ Consequently, the activated cells were observed in abnormal tissue of almost all brain diseases, including various neurogenerative disorders, including multiple sclerosis (MS), Parkinson disease (PD), epilepsy, Alzheimer disease (AD), HIV-associated dementia,^40^ neuronal damage and inflammation.^42^

 Activated microglia often led to elevated *de novo* TSPO expression, which had long been a biomarker of neuronal damage.^43,44^ The concentration seemed in line with the activation state of the microglia and astrocytes during the progression of the pathology events.^45^ Emerging evidence also indicated that the existence of TSPO had been found in a diverse number of cancerous cells in humans, such as brain, oral, prostate, oesophageal cancers, colon, breast, and ovarian cancers, as well as endometrial and hepatic carcinomas.^6^

 TSPO gene was situated on chromosome 22q13.3 and contained four exons, encoding 169 amino acid residues. Furthermore, its receptor was produced by an alternative splicing variant, namely PBR-S lack exon 2, containing an open reading frame that differed from the TSPO.^46^ A current investigation showed that a single-nucleotide polymorphism in exon 4 predominantly affected ligand binding. Based on previous reports, there were two different types of TSPO, coded by the rs6971 single nucleotide polymorphism (SNP), which was first revealed by a PET study using [^11^C]PBR28.^47^

 TSPO was a well-conserved protein with 169 amino-acid residues folded into five trans-membrane helical structures and constructing a hetero-oligomeric complex with the 30 kDa adenine nucleotide translocase (ANT) and the 32 kDa voltage-dependent anion channel (VDAC), which both constituted the mPTP.^48,49^ Moreover, its structure was composed of an integral membrane protein built by the five transmembrane alpha-helices, namely two intramitochondrial loops, two extramitochondrial loops, one extramitochondrial C-terminal, one intramitochondrial N-terminal, and one cholesterol-binding domain.^50^ Previous studies reported the occurrence of TSPO as a monomer, but current investigations suggested that it could generate oligomeric aggregates with itself and other proteins. There was an indication that it could transform into homo-oligomers to provide a binding site for cholesterol.^16,51,52^ Another research on TSPO C-terminal peptide (amino acid sequences 144-169) by nuclear magnetic resonance showed that the helical conformation for the L144 to S159 fragment was important for cholesterol binding.^53^

 TSPO was involved in several substantial cellular mechanisms, such as steroidogenesis, cholesterol transport, inhibition of ROS, regulation of immune functions, induction of apoptosis, mitophagy, heme synthesis, porphyrin transport, and stress sensing.^1,2,54,55^ Previous studies also showed its occurrence in the peripheral tissues, heart, steroid-producing cells, lung, kidney, and immune system. In CNS, the protein was expressed in lower concentrations and mainly found in the olfactory bulb non-parenchymal regions, including the choroid plexus and ependyma.^44,56^ Although its expression was primarily observed in the outer mitochondrial membrane, it had also been found in red blood cells, which were devoid of mitochondria.^57^ From the cytosol perpendicular to the mitochondria, the five transmembrane domains (TMs) of mammalian or bacterial TSPO protein were tightly linked in the clockwise order of TM1-TM2-TM5-TM4-TM3.^1^

 Activated microglial with the concomitant increase of ligand binding site (i.e., (*R*)-PK11195) post nerve injury could either be seen as a retrograde or anterograde. Trans-synaptic microglial activation was not observed in acute brain disorders of common animal models. Nevertheless, it could be in line with human brain disorders when pathological conditions persevered for years.^58^ Bolmont and colleagues showed that an increase in the accumulation of these cells around the amyloid deposits, and the production of cytokines promoting neuronal death were found in AD pathogenesis.^59^ Therefore, it had been hypothesized that microglia dysfunction played a critical role in amyloid plaque deposition.^39^ TSPO structure provided insight and fundamental information regarding its function and interaction with several ligands.^60^

**Figure 2:**
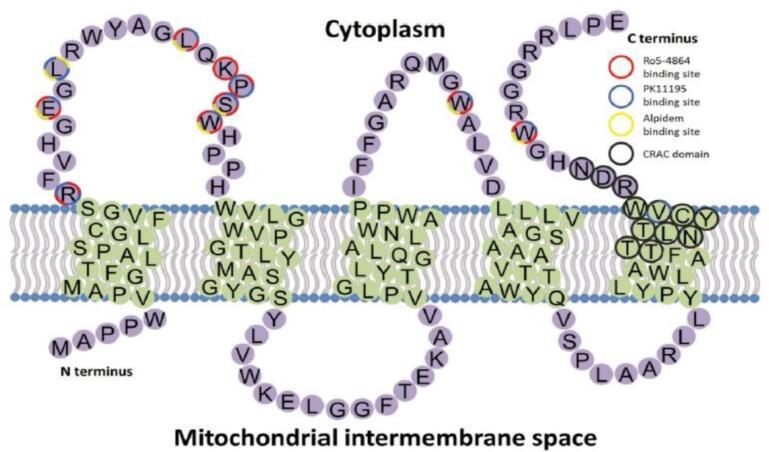
The topology structure of TSPO and ligands binding sites. Reproduced with permission from Liu et al.^44^ Copyright © 2014 John Wiley & Sons, Inc

 A topological model of ligand-TSPO binding sites predicted using molecular modeling and structure-activity relationships analysis suggested that a specific ligand could attach to the targeted amino acid sequences. Based on these analyses, PK11195 interacted with arginine, leucine, glutamic acid, serine, proline, and tryptophan residues, in the form of a key that fitted into a lock, as shown in Figure 2.

## TSPO in cancers

 Over the years, TSPO had become a target of interest for various pathological events, such as brain diseases, anxiety disorders, mood disorders, stress, and neurodegenerative diseases.^9^ However, more up-to-date studies showed that the protein played a major function in the development of various cancers. This indicated that its upregulation within cancerous tissues had attracted considerable interest for potential as a molecular target for diagnostic and chemotherapy purposes.^61^

 TSPO was located in the outer membrane of mitochondria and known as the housekeeping gene. Several reports showed its association with growth factor receptor genes, which were involved in cell proliferation with anti-apoptotic properties. The presence of TSPO also correlated with the level of cholesterol inside the tumor cells. A previous study linked the cholesterol level with the potential of membrane biogenesis, cell energy, and gene expression. The epigenetics mechanism also had a crucial role in TSPO expression, specifically for breast and thyroid cancers. TSPO from the outer part of mitochondrial membranes had an interaction with cytosolic chaperones (HSC70 and HSP90) and Metaxin 1. The various binding sites of TSPO gene promoters had an essential function as transcription factors and signal transducers.^11,62,63^

 The upregulation of this protein in cancer cells was closely related to apoptosis, as shown in previous studies. Furthermore, it interacted with several proteins in the outer and inner mitochondrial membranes by regulating the mitochondrial permeability transition pore (MPTP), which played an essential role in cancer metabolism. MPTP had been reported to have a function in the physiology of Ca^2+^ ROS homeostasis and cell death.^64,65^ The implication of TSPO in cell death had been identified in hepatocellular carcinoma. It also played an inhibitory role in the ferroptosis process through Nrf-2-mediated upregulation of antioxidant gene expression and an increase in the antitumor immunity mediated by CD8 + T cells for immune evasion. The inhibition of TSPO could lead to mitochondrial damage and dysfunction.^66^ In this section, TSPO involvement in some cancers was briefly discussed below.

## Brain cancer

 TSPO was upregulated in various neuropathologies conditions, such as neurodegenerative diseases and gliomas, as well as in different types of brain injury and inflammation.^16^ The expression of the protein was often low in normal brain tissue, while its overexpression was directly related to malignancy grade. Consequently, several TSPO-targeting ligands had been developed for brain cancer imaging or therapy.^17^ Betlazar et al investigated its cellular sources and regional accumulation in the normal mouse brain and discovered a significant level in the glomerular and olfactory nerve layers of the olfactory bulb and the choroid plexus. Persistent expression was also found in neurogenic regions, while low levels were observed in areas of known neurogenesis and cerebellar Purkinje cells.^67^

 A study investigated the role of TSPO genetic variant, namely the rs6971 polymorphic variant, and its correlation with the outcome of glioblastoma patients. The results suggested that the rs6971 gene was a valuable predictor of survival time in glioblastoma patients.^68^ Extensive studies in glioblastoma showed that TSPO was responsible for the generation of ROS in glioblastoma, leading to higher levels compared to non-cancer surrounding cells. Moreover, it was also able to regulate gene expression and cellular energy. TSPO had been studied for its potential as a target for glioblastoma therapy, such as the use of TSPO ligands.^8^ Several molecules were currently being developed for TSPO imaging in brain tumors. The most widely studied third-generation ligands for brain diseases, namely [^18^F]-GE-180, had been involved in clinical PET studies for glioblastoma. However, current evidence suggested that this radioligand exhibited various limitations in *in vivo* applications.^69^

## Breast cancer

 Bhoola et al observed TSPO mRNA in malignant and normal breast tissues. In healthy cells, it was present in several locations, including the cytoplasm of the luminal layer of cuboidal epithelial cells, nuclei of the endothelial cells of vascular tissue located in the intralobular stroma, the outer layer of the discontinuous epithelial cells of terminal ducts and alveoli, nuclei and cytoplasm of lymphocytes, and collagen fibers of fibroconnective tissue.^6^ In *vitro* study on mammary epithelial MCF10A acini (normal) cells showed that TSPO was associated with cancer invasiveness by promoting cell migration and proliferation during mammary epithelial morphogenesis, as well as facilitated partial resistance to luminal apoptosis, thereby enhancing tumor growth.^70^ Enhanced levels were positively associated with breast cancer invasion and shortened disease-free survival in lymph node-negative patients.^71^ Higher levels of TSPO were observed in the estrogen receptor (ER)-negative breast tumors than in ER-positive tumors. An immunohistochemical study by Galiègue et al in normal and breast cancer tissues showed a great increase in its concentration in tumor tissues compared to healthy breast cells. The observation results showed that the expression level was positively linked with Ki-67 and negatively associated with estrogen receptor status.^72^

 A study on its occurrence in neoplastic cells and tumor macrophages of mouse xenografts of human breast tumor cell lines showed that TSPO concentration in various cell types within the tumor contributed to the total TSPO expression. This study also suggested that imaging TSPO in peripheral tumors was feasible due to its overexpression.^73^ Preclinical studies in mice also showed that its overexpression was associated with the degree of breast malignancy. During cancer cell proliferation, TSPO accumulated in the nucleus (Figure 1). The binding capability of the protein also increased in the cancerous cells at the center of the tumor mass. According to a previous study, TSPO played a major role in apoptosis and chemosensitization in cancer cells, making it a suitable target for drug development aimed at developing new therapeutic strategies and a biological marker for cancer imaging.^74^

## Melanoma

 According to previous studies, skin cancer was characterized by abnormal skin cell growth and could be categorized into two groups, namely keratinocyte cancer, also known as non-melanoma skin cancer, and skin melanoma.^75^ The incidence of melanoma continued to increase globally and had the highest mortality percentage among the 2 categories.^75,76^ Despite its low incidence of less than 5%, it had a mortality rate above 10%. Melanoma was also known as one of the most aggressive cancer due to its high capability for metastasis, which was the leading cause of mortality with a 5-year survival rate of approximately 13%.^76^

 The mitogen-activated protein kinase (MAPK) signaling was one of the critical pathways, which served as the main regulator in melanoma development, and its activation was registered in 90% of skin melanomas. Furthermore, the signaling cascade was often activated by mutations in the B-raf proto-oncogene gene, leading to the activation of cell proliferation or NRAS.^76,77^ The MAPK signaling pathway played an important role in transporting extracellular signals, including hormones and key regulatory proteins to the cell nucleus, enabling the expression of genes for cell proliferation and differentiation.^76^ Mutagenic activation of MAPK signaling was a main event underlying tumor progression, and its signaling was known to be triggered by protein kinase C, which modulated the expression of TSPO.^77^ A study showed that there was an increased level of TSPO protein in differentiated cells (melanoma) compared to normal cells.^6,77^ Ruksha et al investigated the role of the protein in melanoma pathogenesis in skin biopsies of patients. A previous study found that TSPO levels correlated to increased tumor invasion levels using immunohistochemistry and real-time PCR.^78^

## Colorectal cancer

 Colorectal cancer (CRC), most often located in the large intestine (colon) or rectum, generally grows slowly from the adenomatous polyp or adenoma. In 2018, the International Agency for Research and Cancer (IARAC) estimated that CRC was the third most prevalent cancer and the second leading cause of death on a global scale.^79^ According to a report, there were 104 610 new cases and an estimated 53 200 deaths in the United States at the end of 2020.^80^ The increased cases of CRC were believed to be associated with changes in modern society’s dietary habits and lifestyle.^81,82^ Consequently, several methods had been developed to detect CRC, including colonoscopy, CT colonography, capsule endoscopy, and the use of specific biological markers. CRC biomarkers were non-invasive assays to detect molecular markers from tissue, blood, stool, and urine.^82,83^

 TSPO expression was detected in healthy colon, but its expression was higher in colon cancer.^84^ In a normal colon, the protein (TSPO mRNA) could be observed in the cytoplasm of the goblet and absorptive cells of Crypts of Lieberkhün, nuclei, cytoplasm of the lymphocytes of lamina propria, and plasma cells.^61^ A previous report involving 55 CRC patients showed that 67% had TSPO protein overexpression in tumor tissues and the level of TSPO-mRNA expression in colon carcinoma was substantially higher compared to rectal carcinoma.^85^ Another study showed that the protein levels were increased in the enterocytes of inflammatory bowel disease biopsies. Based on immunohistochemical data on normal colonic mucosa, its immunoreactivity was only detected on the surface of epithelial cells, and enterocytes highly expressed TSPO, while its expression was not detectable in the goblet cells. This finding indicated that TSPO could serve as a marker of the repair process in inflammatory bowel diseases.^86^ Berroterán-Infante et al showed that [^18^F]-FEPPA (*N*-acetyl-N-(2-[^18^F]-fluoroethoxyben-zyl)-2-phenoxy-5-pyridinamine) had a high binding affinity to TSPO in CRC, as suggested by a competitive binding assay, and could have potential to be translated into clinical CRC imaging using PET.^87^

## Lung cancer

 In normal lung tissues, TSPO mRNA was observed in the plasma cells, the cytoplasm of the macrophages, fibroblast in the stroma area, smooth muscle fiber surrounding the pulmonary artery, and cuboidal cells that lined the respiratory bronchiole. It was also found in the nuclei and cytoplasm of the endothelial cells of the pulmonary artery and lymphocytes in the surrounding stroma of normal lung tissue.^6^ Furthermore, its expression was observed in H1299 cells after exposure to cigarette smoke,^88^ and it was suggested to be associated with cigarette smoke-induced cytotoxicity, which could lead to oral and pulmonary ailments as well as lung cancer.^89^

 Zhang et al explored the potential of TSPO targeting PET radiotracer [^18^F]-PBR06 compared to [^18^F]-FDG for differentiating inflammation and lung cancer in mice. The study showed that [^18^F]-PBR06 uptake in the area of inﬂammation was greatly higher compared to lung cancer tissues, while [^18^F]-FDG showed increased accumulation in both peripheral lung cancer and inflammatory nodules. Despite being out of expectations, this result suggested that [^18^F]-PBR06 PET/CT imaging could be considered an inﬂammation-specific PET imaging tracer for pulmonary nodule detection.^90^ According to Hatori et al, TSPO PET tracer, namely [^18^F]-FEDAC was used to visualize lung inflammation in acute lung injury rats. This study showed that increased TSPO expression (confirmed with Western blot) corresponded to increased lung uptake of [^18^F]-FEDAC. Compared to the control, a substantial increase in the protein level was found in the inflamed lung tissues. Moreover, it was observed that [^18^F]-FEDAC uptake was specific to TSPO elevation with the stimulation of macrophages and neutrophils in the lung. Based on this result, improved TSPO levels correlated with the advance of lung inflammation.^91^

 PET imaging in normal rats showed a high and specific *in vivo* binding in TSPO-enriching organs, including the adrenal gland, lung, heart, and kidney. Zeineh et al investigated the protective effect of TSPO ligands MGV-1 and 2-Cl-MGV-1 against cigarette smoke-induced cellular toxicity in H1299 lung cancer cells. The results showed that pretreatment with the ligands at 24 h before cigarette smoke exposure differentially modulated the cellular insult and cell arrest in H1299 lung cancer cells through the inhibition of ATP synthase reversal, ROS generation, depolarization of the mitochondrial membrane, and increased levels of LDH. Therefore, treatment with MGV-1 and 2-Cl-MGV-1 was helpful in preventing TSPO-associated lung cancer.^92^

## Prostate cancer

 Prostate cancer had become one of the leading causes of mortality among men.^93^ Furthermore, there were an estimated 1.4 million new cases and 375 000 deaths worldwide.^94^ According to previous studies, prostate cancer growth and progression was a complicated process. The androgen signaling system, as well as its interactions with other routes, influenced cellular processes ranging from growth, differentiation, and cell cycle to growth arrest and death. Furthermore, cells often became due to adaptation and modification. Prostate cancer was androgen-dependent and relied on the androgen receptor (AR) to mediate the actions of androgens, with the AR being expressed in development.^95^ Androgen deprivation treatment had been used to decrease androgen-dependent prostate cancer cell proliferation, mainly in the metastatic stage. Although in the initial stages of treatment, the majority of patients suffered from hormone-refractory cancer, also recognized as castration-resistant prostate cancer (CRPC). This condition continued to grow even though the patients were undergoing therapy. In recent studies, it was discovered that all types of prostate cancer, including androgen-dependent, androgen-independent, and CRPC, exhibited TSPO expression.^96^ Therefore, the expression level could be used to evaluate the course of prostate cancer and the development of other cancers connected to its therapy.

 TSPO mRNA was found in the cytoplasm of the inner columnar epithelium, the cytoplasm and nuclei of the outer cuboidal, and the nuclei of fibroblasts of the fibromuscular stroma in normal prostate tissue. It was also discovered in the cytoplasm of cancer cells growing in nests or sheets of Grade III adenocarcinoma of the peripheral duct and acini.^6^ TSPO was primarily important for transporting cholesterol throughout the outer mitochondrial membrane for cell signaling and steroid production.^93^ The levels of the protein were elevated in prostatic intraepithelial neoplasia, primary and metastatic prostate cancer, but lower in benign prostatic hyperplasia and normal prostate cells.^97^ Based on findings, TSPO-targeting PET ligand uptake was higher in tumors compared to normal tissue, implying that using the ligands could improve prostate cancer detectability, accuracy, and characterization.^93,97,98^

 Several types of TSPO-targeting PET ligands had been produced with promising success. The majority of the radiotracers studied for use in PET imaging were either ^11^C or ^18^F labeled.^99^ For example, the absorption of [^18^F]-fluciclovine in prostate cancer cells was found to be increased in patients with recurrence of the condition prostate cancer. Another TSPO radioligand, [^18^F]PBR316, showed tumor uptake of 1.67 ± 0.43% ID/g in mouse model at 1 h after injection with modest binding sensitivity to human single nucleotide polymorphism rs6971.^97^ Other promising candidates with the capacity to detect prostate adenocarcinoma include [^18^F]-fluorothymidine ([^18^F]-FLT), [^18^F]-NaF, [^18^F]-F-choline, and [^18^F]-BAY 864367.^99^

## Radiolabeled TSPO ligands: A brief overview

**Figure 3:**
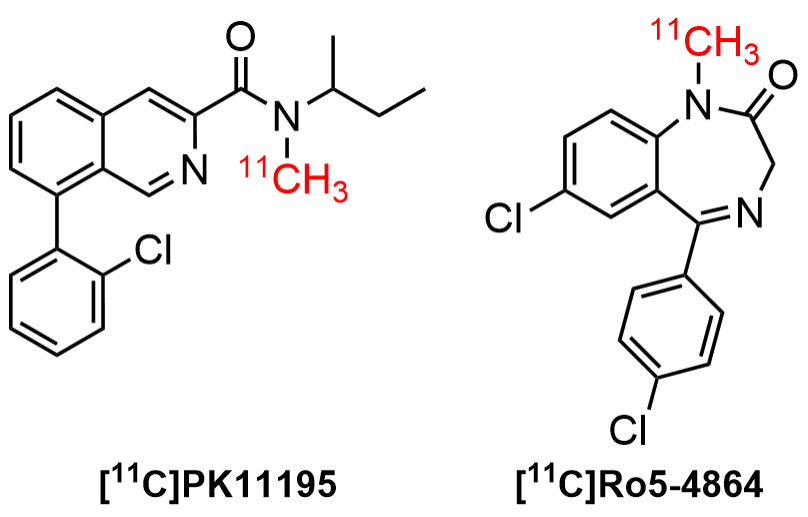
First generation radiolabeled TSPO ligands

**Figure 4:**
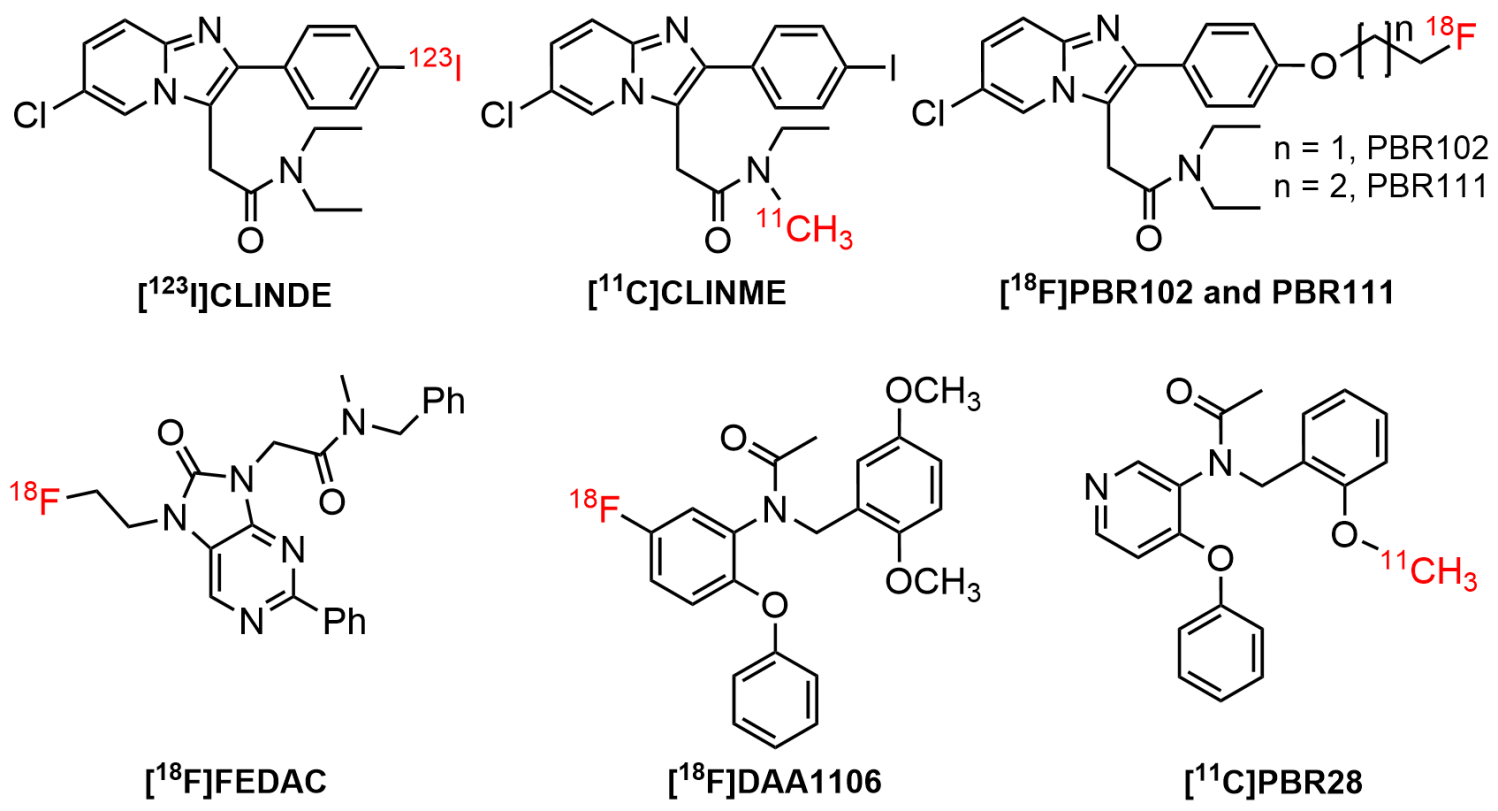
Second generation radiolabeled TSPO ligands

**Figure 5:**
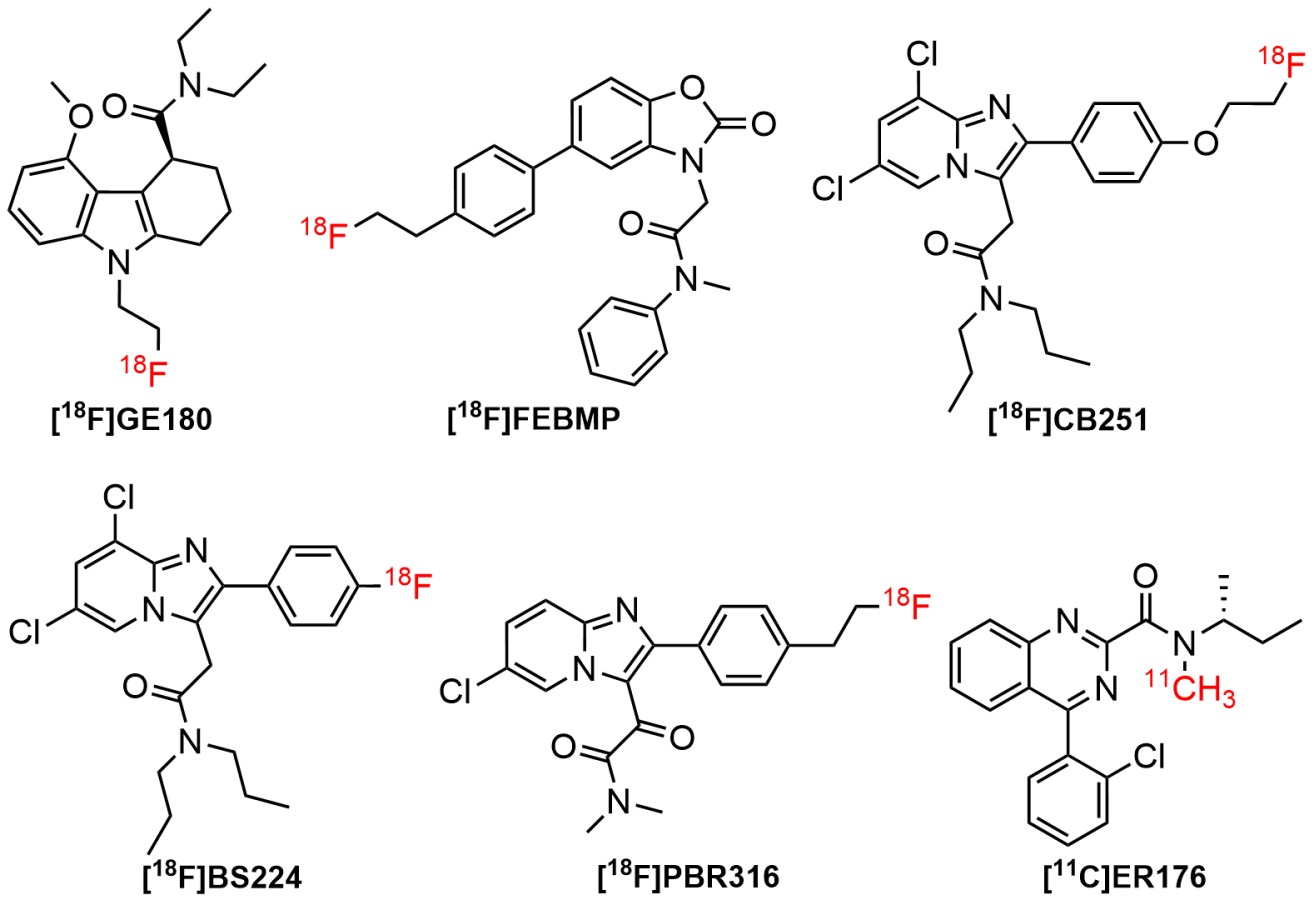
Third generation radiolabeled TSPO ligands

 The use of SPECT/PET tracers for TSPO imaging had grown widely over the last three decades. Furthermore, almost all the physiological processes occurring in the biological system were related to the interaction of cellular receptors with small and large molecules. One of the interesting findings in the pharmacology arena was the interaction of active receptor sites with a wide range of small ligands. For instance, the contact of TSPO receptor with a diverse number of ligands in brain diseases. Studies suggested that TSPO was bound to putative endogenous ligands, such as protoporphyrin IX and cholesterol.^100-102^ It has also been shown that it bound to a wide type of synthetic compounds, including (1) first-generation radiotracers, such as isoquinoline carboxamides ([^11^C]PK 11195^103^) and benzodiazepines ([^11^C]Ro5-4864^5^) (Figure 3); (2) second generation radiotracers, such as imidazopyridines ([^123^I]CLINDE,^104^ PBR-102 and PBR-111,^105^ and [^11^C]CLINME),^5^ 2-aryl-8-oxodihydropurine acetamides ([^18^F]FEDAC),^106^ phenoxyarylacetamides ([^18^F]DAA1106),^107^ aryloxyanilides ([^11^C]PBR28),^108^ (Figure 4); and (3) new generation TSPO radiotracers, such as trycyclic indoles ([^18^F]GE180)^109^ (Figure 5). These radioligands had been employed in various human disease studies, particularly for the visualization of cancers and neuroinflammation.

 Benzodiazepines (benzos) were a class of important drugs containing a benzene ring and a diazepine moiety. These drugs had been used as anticonvulsants, muscle relaxants, sedative-hypnotics, and anxiolytics in CNS-related disorders. The biological effects could be attributed to the mediation of central benzodiazepine receptors (CBRs) in the CNS.^110^ Furthermore, they specifically acted as a positive allosteric modulator on the CNS neurotransmitter, namely gamma amino butyric acid (GABA)-A receptor.^111^ A well-known benzodiazepine compound, Ro5-4864 (4’-chlorodiazepam) was the first compound used to distinguish TSPO from CBR. A study of [^11^C]Ro5-4864 in human subjects with brain cancer exhibited a high non-specific binding and a low affinity in brain.^5^ It also found that Ro5-4864 induced cell proliferation in breast epithelial cells.^112^ Moreover, [^11^C]Ro5-4864 exhibited neuroprotective properties to prevent β-amyloid deposits in animal model of neurodegenerative diseases.^12^

*N*-butan-2-yl-1-(2-chlorophenyl)-*N*-methylisoquinoline-3-carboxamide (PK11195), a classic isoquinoline carboxamide ligand, was the first high-affinity nonbenzodiazepine ligand to bind selectively to TSPO receptor. Consequently, it was broadly used alongside Ro5-4864in animal models and humans with CNS diseases. The radiolabeled version of the ligand, [^11^C]PK 11195 was initially used for human glioma imaging in 1989.^113^ The PK11195bound to TSPO, specifically in peripheral organs and hematogenous cells linked to γ-aminobutyric acid (GABA)-regulated channels, as well as the active site of microglia. The binding capacity of [^11^C]PK11195 in patients with AD and MS were correlated with the activation of microglia. This indicated that TSPO could be a clinically important molecular target for imaging diseases by PET.^58^ The binding sites of PK11195were also observed in non-mitochondrial fragments of the brain and mitochondrial-free erythrocytes.^114^ Awad and Gavish showed that ithad a greater binding affinity to TSPO compared to Ro5-4864 in human cerebral cortex and kidney membranes.^115^ A study using radiolabelled ligand [^3^H]-PK11195 showed an affinity of PK11195was 9.3 nM (*K*_i_). Moreover, PK11195exhibited good selectivity, as suggested by its low activity in several molecular targets, including central benzodiazepine, benzodiazepine, GABAergic, catecholaminergic, and opiate receptors.^100^

 Over the years, [^11^C]PK11195 had been employed for in *vivo* PET imaging of animal models and human subjects with neurodegenerative disorders, such as AD, MS, PD, Huntington disease, frontotemporal disorders, and amyotrophic lateral sclerosis. However, the use of the radiolabeled PK11195 had shown varying levels of success. Although some potential *in vitro* data, the low signal-to-noise ratios and short half-life (20.4 minutes) had limited its application in in *vivo* imaging studies in recent years.^113,116^ This prompted to the development of second-generation TSPO-selective ligands bearing various structural classes for activated microglia imaging by PET and SPECT.

 The radiolabeled imidazopyridine derivatives, including [^123^I]-CLINDE, had raised substantial interest as it displayed a favorable binding for TSPO. *In vitro* binding studies in the kidney, adrenal, and cortex mitochondrial membranes showed that the ligand bound to TSPO with affinity values of 12.6, 0.20, and 3.84 nM (K_d_), respectively.^117^ Meanwhile, another imidazopyridine radioligand, [^11^C]-CLINME, was reported to have an improved imaging results than [^11^C]PK11195in rodents-induced with local acute neuroinflammation.^5^ The binding affinity of [^11^C]-CLINME was found to be comparable with [^11^C]-PK11195 and [^18^F]-PBR111.^118^

*N*-(2,5-dimethoxybenzyl)-*N*-(5-fluoro-2-phenoxyphenyl)acetamide ([^18^F]DAA1106) had received intensive attention due to its superior affinity for TSPO as well as selectivity over CBR. In a preclinical binding study, DAA1106showed an affinity (IC_50_) of 0.28 nM.^119^ It also possessed low affinity (IC_50_ = 10 000 nM) for other receptors, such as GABA_A, _Kappa_1_, and central benzodiazepine receptors, as well lower affinities (IC_50_ > 10 000 nM) for 54 different proteins, covering receptors, ion channels, second messengers, and uptake/transporters.^107^

*N*-(2-methoxybenzyl)-*N*-(4-phenoxypyridin-3-yl)ethanamide (PBR28) had been used in *vivo* to examine a variety of CNS pathologies, such as AD, epilepsy, and MS.^120^ PBR28 seemed to possess an improved signal-to-noise ratio and reliability compared to the first generation (R)-[^11^C]PK11195 tracer.^121^ Despite the ability of this ligand to displace PK11195, the assessment of the in *vivo* binding had been hampered by the absence of a valid reference region.^122^ Radioconjugate PBR28 analog, [^11^C]ER176 exhibited four times higher binding potential relative to nondisplaceable uptake (BP_ND_) for high-affinity binders compared to [^11^C]PBR28.^123,124^ In 2008, Fookes et al introduced a novel series of TSPO ligands bearing imidazopyridine moiety, namely 2-(6-chloro-2-(4-(2-fluoroethoxy)phenyl)imidazo[1,2-a]pyridin-3-yl)-*N*,*N*-diethylacetamide (PBR102) and 2-(6-chloro-2-(4-(3-fluoropropoxy)phenyl)imidazo[1,2-a]pyridin-3-yl)-*N*,*N*-diethylacetamide (PBR111) for visualization of TSPO. All ligands contained fluorine in their structures, and this led to the suitable radiolabelling with fluorine-18. The derivatives were found to be potent and selective with K_i _of 3.3-5.8 nM and 800-above 5000 nM for TSPO and CBR, respectively.^105^

 Flutriciclamide ([^18^F]GE180), the third generation of TSPO tracer showed high signal in the inflammation site, low non-specific binding, and low radiometabolites signals in animal models of middle cerebral artery occlusion and AD. According to a previous study, it seemed to provide better imaging properties compared to [^11^C]PK11195 and [^18^F]DPA714. [^18^F]GE180 displayed an improved binding capability to TSPO in both rat and human subjects with *K*_i_ values of 0.87 nM and 9.2 nM, respectively.^17,125^ Nevertheless, in in *vivo* studies on MS patients, it demonstrated a low volume of distribution, indicating limited brain penetration. This led to the production of an image quality that was insufficient to visualize the differences in binding to low-affinity binders and high-affinity binders.^17^ Apart from [^18^F]GE180, several third-generation TSPO radioligands based on various structural platforms had been developed, including [^18^F]FEBMP,^126^ [^11^C]ER176,^127^ [^18^F]BS224,^128^ [^18^F]CB251,^129^ and [^18^F]PBR316^97^ (Figure 5).

## New perspective: Image-guided surgery targeting TSPO

**Figure 6:**
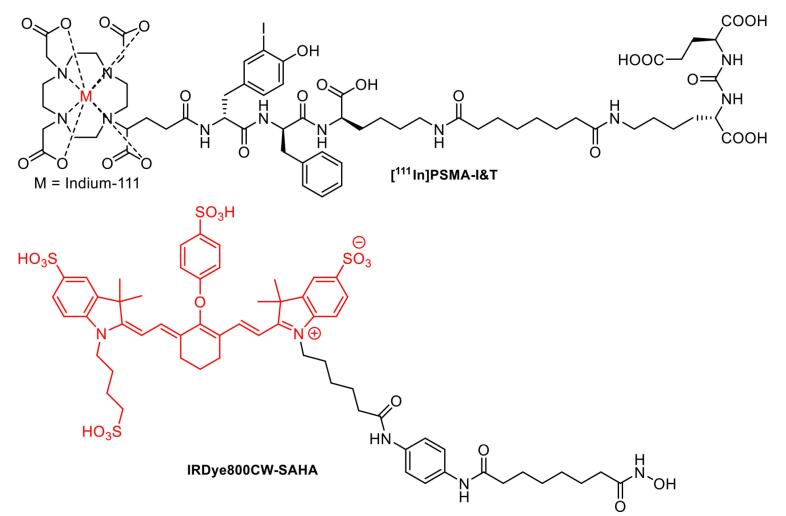
Structure of [^111^In]-PSMA-I&T and IRDye800CW-SAHA

 Extensive efforts had been made to identify novel, rapid, and accurate intraoperative margin measurement tools, which were currently advanced to clinical trials. One of the most crucial aspects of cancer surgery was the complete removal of tumor tissue to prevent recurrence, leading to improved survival of patients. RGS had long been used to enhance the complete resection of tumors (reviewed in^21,26^). For instance, [^111^In]-PSMA-I&T (Figure 6) represented a valuable agent for the intraoperative detection of small tumor lesions and had entered clinical trials on prostate cancer patients. Preoperative SPECT/CT visualization and radio-guided removal of PSMA-positive lesions using the compound had also been successfully demonstrated.^130^ Furthermore, FIGS was among the biophotonic-based techniques, which had been studied for its potential in tumor surgery.^131,132^ Several fluorescent probes were developed for FIGS, such as IRDye800CW-suberoylanilide hydroxamic acid (SAHA) (Figure 6). The probe exhibited rapid tumor accumulation and was successfully used for the resection of orthotopic hepatocellular carcinoma in a mouse tumor model during the FIGS experiment.^133^ Several other fluorescent probe candidates built from various structure classes had been investigated in *in vitro* and *in vivo* settings.^134,135^

 Over the years, multimodal/hybrid tracers had been widely used in non-invasive molecular imaging investigations. Multimodal tools could provide preoperative information, both anatomical aspects using ultrasound, computed tomography (CT), and magnetic resonance imaging (MRI), as well as functional aspects using PET, SPECT, or optical imaging (fluorescence imaging) during the surgery. However, SPECT and PET had dominated the imaging modalities in clinical settings in recent times. Recent studies also integrated these tools with other modalities, including CT and MRI. The limitations of radiotracers application in preoperative and intraoperative surgery, particularly the inability to perform visual confirmation of the tissue were the driving force for the development of more advanced hybrid technologies, such as dual-imaging (hybrid probes) by combining radioactive and fluorescent probes.^136^ The use of radioisotopes integrated with the fluorescence agent could ensure synergistic capabilities during intraoperative target delineation.^137^

 The hybrid probes (i.e., radio-fluorescent molecules) could be synthesized by combining radiotracer-attached molecules with various fluorescence compounds, such as small dyes and nanoparticles. Upconversion and downconversion nanoparticles (UCNPs and DCNPs) were two types of fluorophore nanoparticles used in image-guided surgery. Cordonnier et al developed near-infrared (NIR)-fluorescence NaYF4:Yb, Tm@NaYF4 core/shell UCNPs modified with KuE ligand. This UCNP exhibited an excellent affinity for prostate cancer both in vitro and in vivo.^138^ Ren et al developed angiopep-2 peptide conjugated DCNPs for imaging-guided surgery of orthotopic glioma. This DCNP was developed with Er-based lanthanide with additional Dye-blush polymer enabling ^4^I_13/2_→^4^I_15/2_ transition in the NIR-IIb fluorescence region.^139^ Apart from UCNPs and DCNPs, there were also several types of nanoparticles developed for similar applications. These included the development of a PEG-based nanoparticle with silicon 2,3-naphthalocyanine core, which had an activatable ability after accumulation in tumor.^140^ Other studies implemented fluorescence gold nanoparticles (AuNPs) which had been modified with glutathione as fluorophore in imaging-guided surgery. Chen Hung-Li proposed aptamer-modified Glutathione-AuNPs as a guided surgery agent with dual imaging fluorescence and computed tomography mode ability for prostate cancer.^141^ Meanwhile, non-modified glutathione-gold nanoclusters (AuNCs) showed high accumulation in bone, making them suitable for bone imaging.^142^ Apart from conventional fluorophore relying on the conversion of NIR and visible photon for activation, there was a combination of CLI with radiolabeled molecules for RGS of cancer. Shi et al developed Eu^3+^ doped gadolinium oxide ­(Gd_2_O_3_:Eu), which could generate optical fluorescence signal when interacting with photon emitted from ^18^F-FDG. This combination of nanoparticles and ^18^F-FDG increased in vivo imaging signal 369 times higher compared to using ^18^F-FDG alone.^143^ Furthermore, it could be considered hybrid probes showed promising characteristics. Several examples of hybrid probes developed based on nanoparticles are presented in Table 1.

**Table 1:** Examples of hybrid probes developed based on nanoparticles

**No.**	**Hybrid Probes**	**Potential Applications**	**References**
1	^64^Cu, 800CW (fluorescence [NIRF] dye), and TRC105 (IgG1 monoclonal antibody) to the surface of MSN (mesoporous silica nanoparticles)	In vivo tumor vasculature targeted PET/ NIRF imaging	^144^
2	^99m^Tc and indocyanine green (ICG) loaded in polyamidoamine (PAMAM) based functionalized silica nanoparticles	Biopsy of the sentinel lymph node and imaging of HER2-expressing cancer cells	^145,146^
3	^64^Cu-doped CdSe/ZnS QDs	In vivo glioblastoma imaging and detection	^147^
4	^64^Cu-labeled DOTA–QD–VEGF	Tumor vasculature	^148^
5	^64^Cu-doped AuNCs	Glioblastoma CRET-NIR and PET imaging	^149^
6	^127^I/^124^I and phenol-substituted analogs of DiD/ DiI on SapC-DOPS nanovesicles	Optical and nuclear imaging of glioblastoma	^150^
7	^89^Zr and Cy5 with ultrasmall cRGDY-conjugated silica nanoparticles (C dots)	Melanoma imaging	^151^

**Figure 7:**
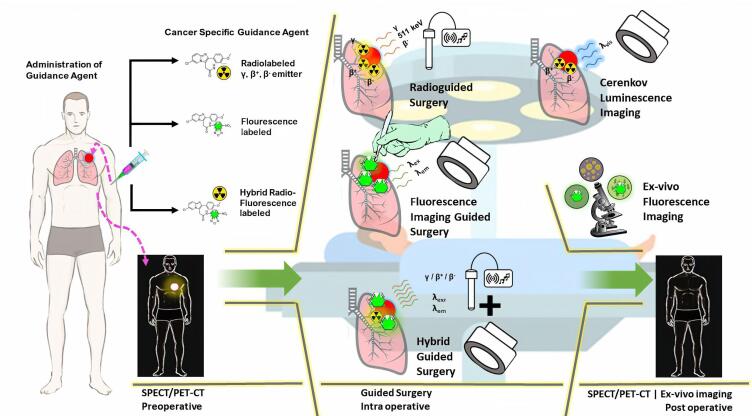
Schematic overview of radio, fluorescence, and hybrid imaging, and their potential use for preoperative, intraoperative, and post-operative cancer surgery. Radioguided surgery (RGS) often uses gamma, beta, or Cerenkov radiation emitted by the radiotracer to navigate tumor location, while fluorescence imaging relies on fluorophore. Hybrid imaging-guided surgery combines a radiotracer and a fluorescence compound to reduce the limitations of each method, thereby increasing the precision of the surgery process.^29,137,152^ Several images were taken from publicdomainvectors.org and openclipart.org under a Creative Commons Attribution CC0 1.0 (Universal Public Domain Dedication)

 The choice of fluorophore molecules was vital to ensure the success of the design of radio-fluorescent probes. For clinical application of tumor surgery, the fluorophore must exhibit good biocompatibility with visible wavelength (400-650 nm) or even NIR wavelength ( > 750 nm), allowing good tissue penetration while preventing the autofluorescence of blood and tissues.^29,69,152^ The primary use of hybrid probes was as surgical guidance, particularly in oncology. RGS was often carried out using specific radiotracers, such as ^99m^Tc-nanocolloid for SNL visualization, and receptor targeting employed ^99m^Tc-PSMA and ^111^In-DOTA-TOC before the development of radio-fluorescence imaging. The combination of fluorescence and radioguided surgery into a hybrid molecule could undoubtedly be used to overcome several limitations associated with the single use of radiotracer or fluorescence imaging (Figure 7), thereby improving the clinical impact of the procedure.^137^ Over the years, several hybrid probes had been developed and shown promising clinical trial results, including [^68^Ga]/[^177^Lu]-PSMA-I&F,^153^ [^18^F]-Cy5-BF_3_,^154^ [^64^Cu]-DOTA-NT-Cy5.5,^155^ [^111^In]-DOTA-girentuximab-IRDye800CW,^156^ and [^68^Ga]-IRDye800CW-BBN.^157^

**Figure 8:**
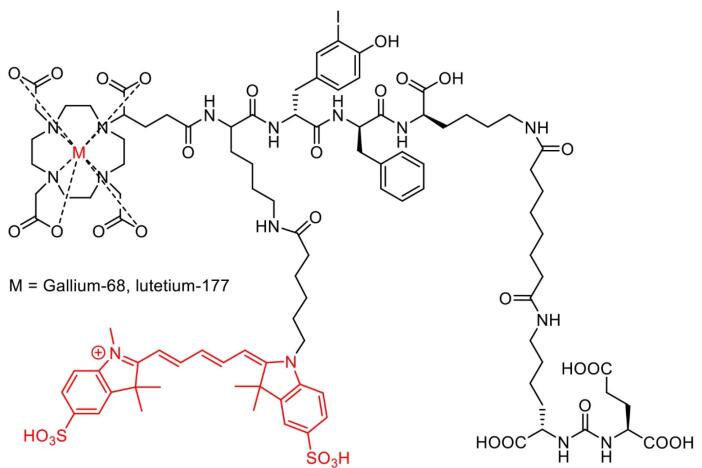
Chemical structure of [^68^Ga]/[^177^Lu]-PSMA-I&F

 [^68^Ga]/[^177^Lu]-PSMA-I&F (Figure 8) exhibited high PSMA-targeting efficiency and favorable pharmacokinetic properties, enabling high-contrast and sensitive detection for *in vivo* study of PSMA expression using preclinical PET/SPECT and optical imaging.^153^ In addition to the hybrid molecules targeting PSMA, various other molecules had been developed for imaging multiple cancer types. For example, [^111^In]-DOTA-girentuximab IRDye800CW showed a good tumor-to-normal kidney ratio (T:N ratio) with no serious adverse effects observed, suggesting a potential use for intraoperative guidance of clear cell renal carcinoma resection in patients.^156^

**Figure 9:**
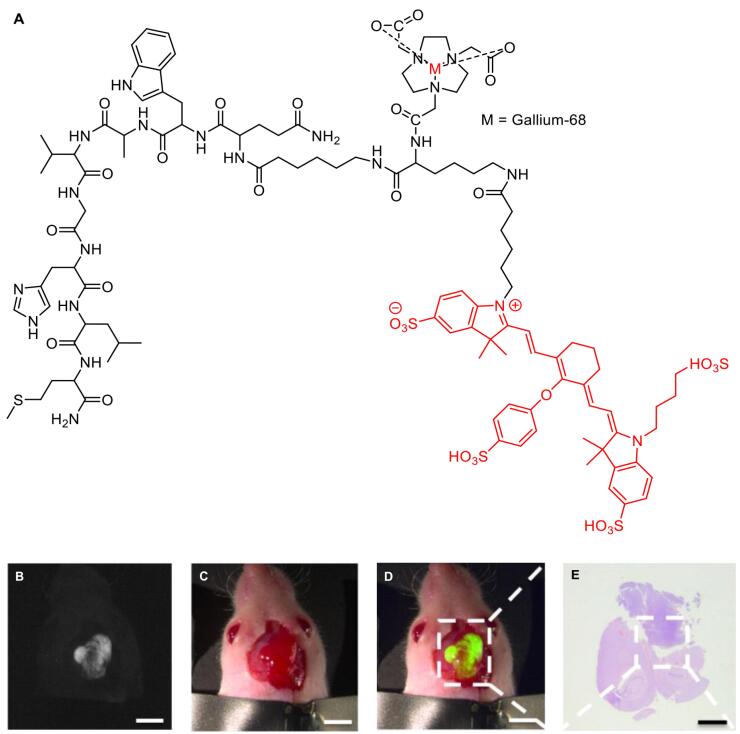
Chemical structure of [^68^Ga]-IRDye800CW-BBN (A). The fluorescence image confirmed the presence of glioblastoma multiforme in nude mouse (B). The color image showed the tumor nodule (C). Merged images of B and C, showing fluorescence signals from tumor tissues (D). The H&E image confirmed the result of tumor (E). Scale bars, preoperative row, 5 mm. Reproduced with permission from Li et al.^157^ Copyright © 2018 Ivyspring International Publisher

 Moreover, [^68^Ga]-IRDye800CW-BBN (Figure 9), a near-infrared fluorescence (NIRF) targeting GRPR in glioblastoma multiforme, had also entered clinical trials for further assessments. Preclinical and clinical evaluations showed that the compound possessed significantly higher tumor fluorescence signals compared to those from adjacent brain tissue, allowing for better intraoperative glioblastoma visualization and safe resection.^157^

**Figure 10:**
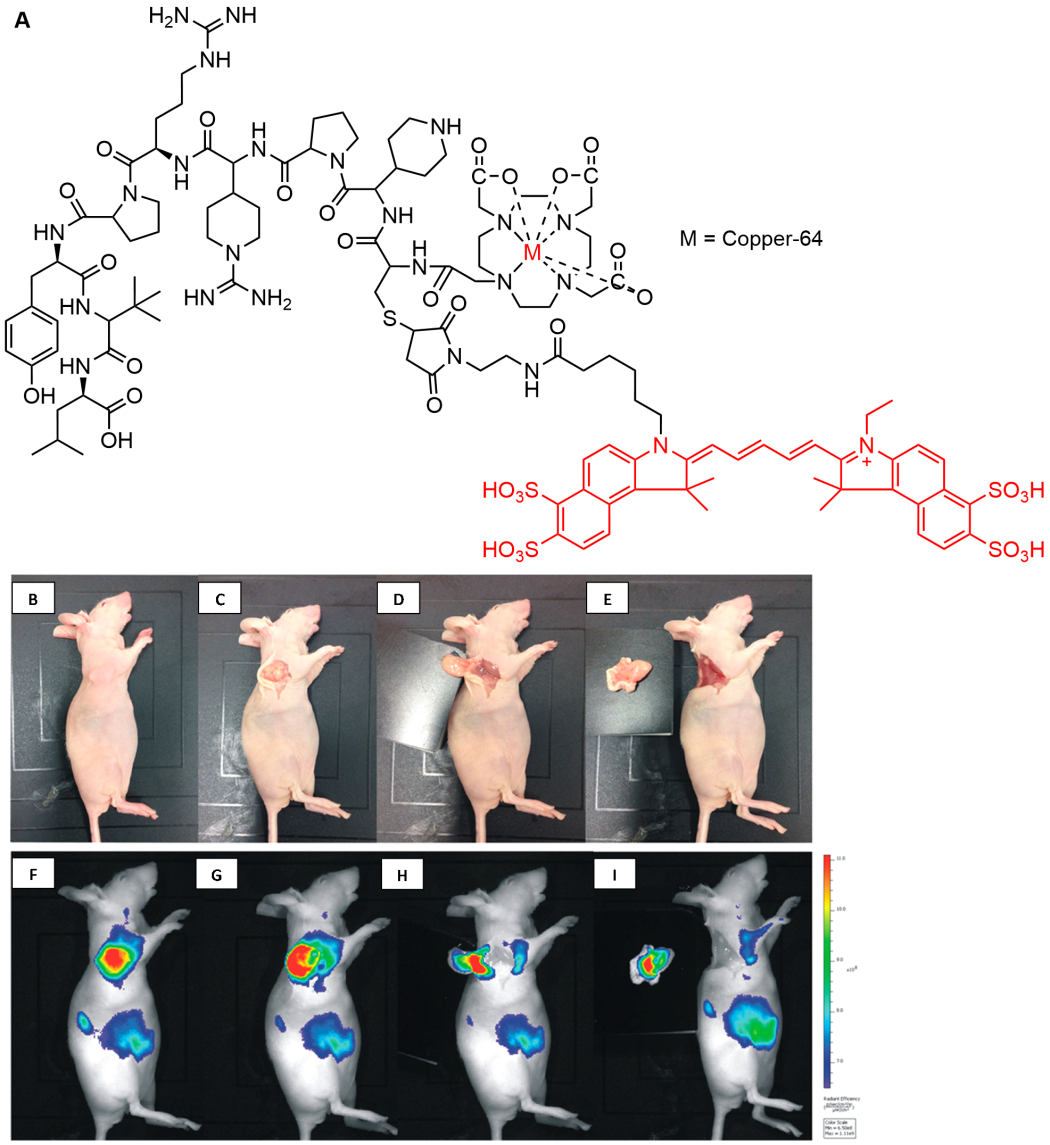
Chemical structure of [^64^Cu]-DOTA-NT-Cy5.5 (A). Digital pictures of the mouse bearing HT-29 tumor after 4 h injection of [^64^Cu]DOTA-NT-Cy5.5 and the tumor removal (B-E), and fluorescence imaging for surgical navigation during surgery (F-I). Reproduced with permission from Deng et al.^155^ Copyright © 2015 American Chemical Society

**Figure 11:**
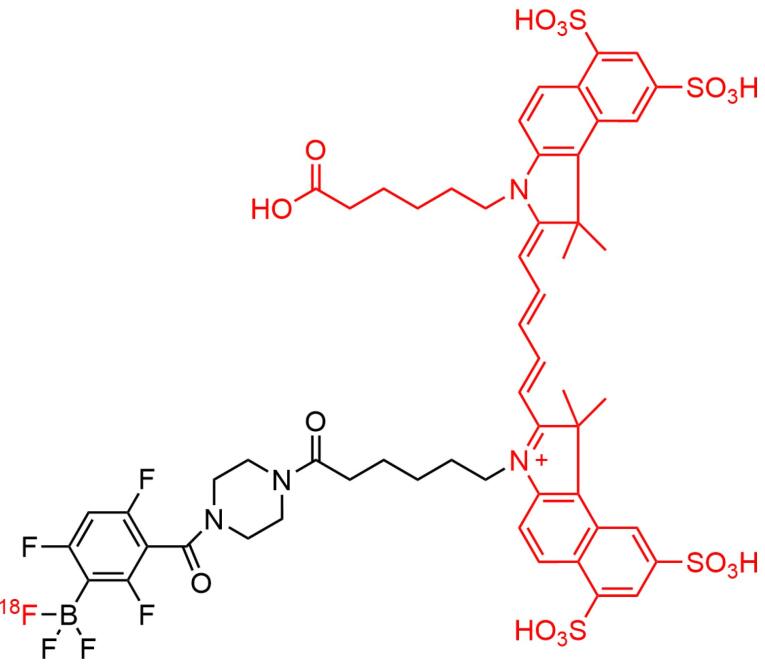
Chemical structure of [^18^F]-Cy5-BF_3_

 Deng et al reported a hybrid PET/fluorescent probe, [^64^Cu]-DOTA-NT-Cy5.5 (Figure 10) as a candidate to image the neurotensin receptor-positive tumor. The agent exhibited promising properties with tumor uptake values of 1.91 ± 0.22 and 1.79 ± 0.16% ID/g after 1 and 4 hours injection, respectively, indicating the potential to provide surgery guidance.^155^ Furthermore, An et al designed a hybrid-modality NIR imaging probe based on pentamethine cyanine structure, namely [^18^F]-Cy5-BF_3_ (Figure 11) for visualization of tumor cells. The probe was found to accumulate in tumor areas, and no cytotoxicity symptoms were observed seen. This evidence indicated the promising use of the material for tumor imaging, FIGS, and post-surgery pathological evaluation.^154^

**Figure 12:**
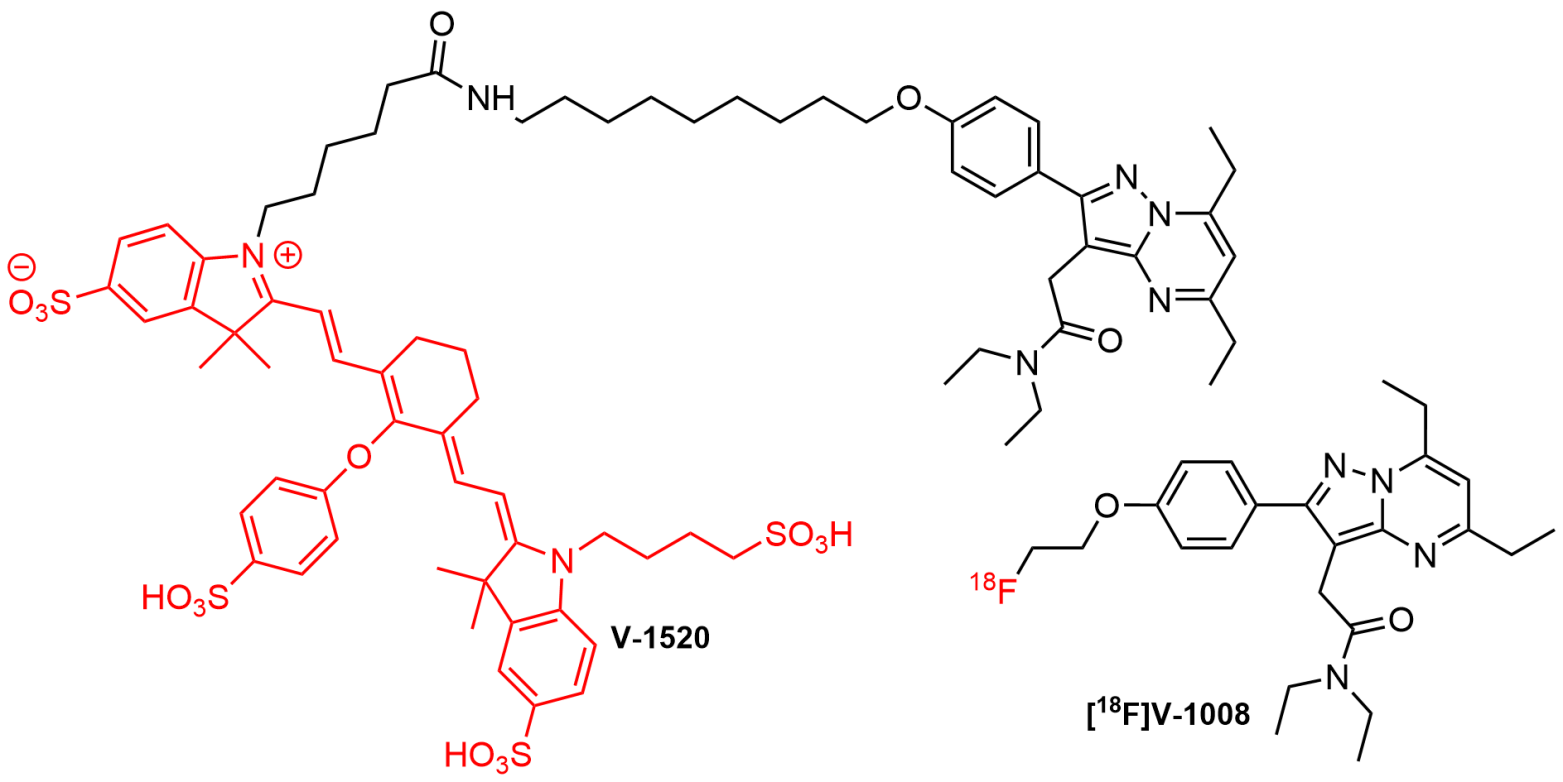
Structure of PET tracer [^18^F]V-1008 and fluorescent probe V-1520

 An early effort to develop specific fluorescent imaging probes targeting TSPO for intended use in FIGS was made by Cohen et al. The study examined TSPO expression in pre-malignant and pancreatic cancer tissues from human samples and genetically engineered mouse tissues utilizing a PET agent, namely [^18^F]V-1008 (Figure 12). Furthermore, the development of TSPO as a molecular target for surgery had also evaluated using a near-infrared TSPO fluorescent probe V-1520 (Figure 12), a compound derived from V-1008 pharmacophore. It was found that [^18^F]V-1008 exhibited good uptake in early pancreatic cancer, while V-1520 could recognize pre-malignant pancreatic lesions and advanced malignant cells, thereby enabling real-time FIGS.^20^ This study paved the way to expand TSPO ligands applications into image-guided surgery practices.

 Although examples of TSPO-targeted molecules for intended use in surgical oncology were rare, it was worth hypothesizing that the available ligands, specifically third-generation types could serve as the molecular templates for the generation of radio-fluorescent compounds. Several developed PET TSPO ligands from various structure classes had been used for studying a wide variety of human diseases. Due to their diverse structures, some of the compounds could also be conjugated with fluorophores after chemical modifications, to generate hybrid probes. It was anticipated that attachment of the bulky fluorophore moiety into the parent ligand influenced its binding capability to TSPO. Based on this result, hybrid probe candidate should be re-examined for its pharmacokinetic properties. In surgical application, the hybrid molecule was first used to navigate the tumor location using SPECT or PET, followed by fluorescence-guided surgical resection.

## Conclusion

 In conclusion, TSPO was an appealing molecular target for various human diseases, including neurodegenerative diseases, inflammation, and cancer. Consequently, several compounds had been developed to target TSPO, and some candidates had entered clinical investigations. In this context, the development of PET/SPECT radioligands to visualize and detect the protein had dominated recent progress in the past few years, as exemplified by the development of various tracers. Based on the advancement of surgical technology in oncology, the future of image-guided surgery appeared to be moving toward the use of hybrid radio-fluorescent probes, which targeted molecular or sub-cellular constituents involved in cancer pathologies. In this review, it was proposed that TSPO could be an alternative molecular target for image-guided surgery of solid cancers, thereby necessitating further investigations in the field of hybrid probes development and biochemistry of TSPO in carcinogenesis. The results were expected to shift the trend in the future from focusing only on TSPO-targeted therapy but also on developing TSPO-targeted hybrid probes for image-guided surgery of cancer.

## Acknowledgments

 The authors acknowledge the use of Servier Medical Art, licensed under a Creative Commons Attribution 3.0 unported license for the partial generation of Figure 1.

## Competing Interests

 The authors declare no conflicts of interest, financial, or otherwise for this study.

## Ethical Approval

 Not applicable.

## Funding

 This study was funded by the National Research and Innovation Agency (BRIN)-Indonesia Endowment Fund for Education (LPDP), Research and Innovation Programme for “Indonesia Maju (RIIM),” (grant number 65/II.7/HK/2022).
